# A 65-Year-Old Woman With No Menopause History: A Case Report

**DOI:** 10.7759/cureus.44792

**Published:** 2023-09-06

**Authors:** Keana-Kelley D Swanner, Larry B Richmond

**Affiliations:** 1 Medicine, Alabama College of Osteopathic Medicine, Dothan, USA; 2 Obstetrics and Gynecology, Regional Medical Center, Anniston, USA

**Keywords:** menopause transition, irregular menstruation, geriatric gynecology, late-onset menopause, geriatric menopause

## Abstract

Menopause is a universal occurrence in a woman's life where menstruation ceases, with an average age of 51.4 years in the United States. Late-onset menopause is defined as menopause after age 55. A thorough PubMed search revealed that there are currently no records of extended cycles through the entirety of a woman’s geriatric years. A 65-year-old G2P2 Caucasian woman was admitted to the emergency department (ER) with a possible cerebrovascular accident. During admission, it was noted that the patient had vaginal bleeding. CT scan revealed a large fibroid, and ultrasound revealed an extremely thin endometrium, excluding endometrial pathology. Gynecology was consulted for post-menopausal bleeding, but in interviewing the patient, she was not surprised at her bleeding. Her LH and FSH levels were low, in the premenopausal range. This is a cautionary tale of an appropriate workup, and the importance of taking a gynecologic history, in the geriatric population.

## Introduction

Menopause is defined as the permanent cessation of menses following at least a six-month time span from the final menstrual period (FMP) and occurs when the ovaries stop producing estrogen. Menopause is a universal process, with the current median age being 51.4 years [1]. The timing of menopause is affected by several factors, including genetics, smoking, and reproductive history. There is considerable variability around the onset of menopause with 5% of women undergoing menopause after age 55 and another 5% between the ages of 40-45 years [2].

Postmenopausal bleeding should be taken seriously secondary to the possibility of endometrial cancer and evaluated thoroughly. Although most causes of postmenopausal vaginal bleeding are benign, the workup includes diagnostic evaluation, including transvaginal ultrasound or endometrial biopsy [3]. In the emergency department, women are usually asked about their last menstrual period (LMP) and current gynecologic symptoms, including bleeding and pelvic pain. A careful history and physical evaluation is crucial in evaluating gynecology patients appropriately and is oftentimes overlooked in the scope of medical evaluations, especially in an emergency department (ER) setting.

## Case presentation

We present the case of a 65-year-old G2P2 woman who presented to the emergency department (ED) with cerebrovascular accident (CVA) symptoms and was admitted and evaluated with pertinent vital signs shown below (Table 1). A gynecological consultation was requested during the hospital stay due to the patient admitting to vaginal bleeding, presumed postmenopausal bleeding. The patient’s past medical history included hypertension, type 2 diabetes mellitus, and stage 3 CKD. The patient had a brief smoking history of five years, but no history of alcohol or recreational drug use.

**Table 1:** Vital signs obtained in the ED

Vital Signs		Reference Range
Temperature (temporal)	97.7 F	97-99 F
Respiratory rate	17 breaths per minute	12-20 breaths per minute
Heart rate	96 beats per minute	60-100 beats per minute
Blood pressure	133/56 mmHg	<135/80 mmHg
SpO_2_	97%	95-100%
BMI	47.6 kg/m^2^	Morbidly obese: >39.9 kg/m^2^

**Figure 1:**
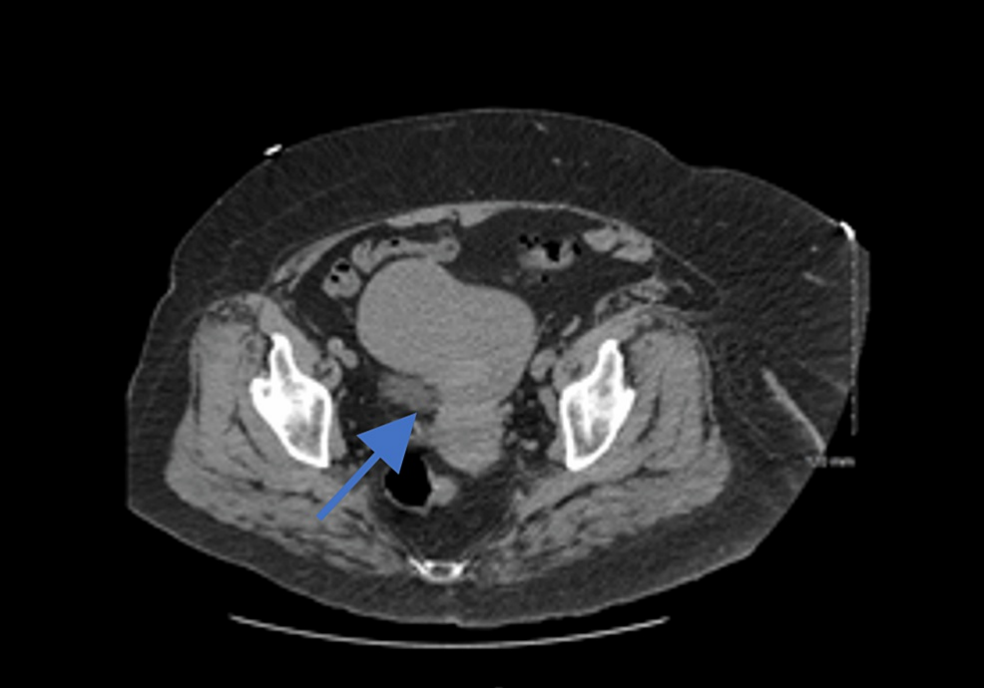
Computed tomography (CT) scan of the abdomen and pelvis without contrast obtained in the ED representing an enlarged lobular appearing uterus with a probable 7-cm right-sided fibroid.

A computed tomography (CT) scan of the abdomen and pelvis without contrast was ordered in the ED and found an enlarged lobular appearing uterus with a probable 7-cm right-sided fibroid (Figure 1). The endometrial lining was not well seen. During the gynecological consultation, the patient claimed that she was still menstruating. Initially a worrisome statement, the patient proved credible as she gave an excellent menstrual history. Furthermore, detailed family history showed late pregnancies and menstruation through the age of 75. A pelvic exam was performed and noted active vaginal bleeding at the cervical os. A transvaginal ultrasound and FSH/LH levels were ordered for confirmation.

Ultrasound confirmed a myoma in the fundus and a thin endometrial stripe, consistent with menstruation. The FSH level was 4.0 IU/L, indicating that the patient was not in the menopausal stage (normal range 4.0-21.5 IU/L). The patient was counseled to remain on aspirin and advised to follow up outpatient with an OB/GYN after discharge for a normal yearly exam.

The patient was discharged on day three of hospitalization to inpatient rehab, with a completely resolved altered mental status and status post-left basal ganglia ischemia of the undetermined age.

## Discussion

In our case, the patient had several risk factors for endometrial hyperplasia, including past history of smoking, morbid obesity, and type 2 diabetes mellitus. This, and her age, led to the assumption that her vaginal bleeding was of pathologic circumstances, instead of possible normal menstruation. A careful history was not taken in the ER, which led to unnecessary testing of the patient. This could have been due to a lack of careful questioning or due to the patient’s altered mental status and inability to give a focused history.

Despite the common name of menopause, there is no single pathway to the end of menses. Menstruation ceases for different reasons, in different ways, at different time points in the lifespan, and is associated with different health risks. Using menopause as a blanket word to describe any “cessation of menses” erases these differences in the physiology, etiology, and health outcomes of the many causes of cessation [4].

With fewer follicles maturing in the aging ovaries, levels of follicle-stimulating hormone (FSH) and luteinizing hormone (LH) become elevated due to disinhibition, although 17β-estradiol (E2) levels become highly variable. On average, the perimenopausal period lasts about four years. After the LMP, both E2 and progesterone remain circulating at very low levels (2-35  pg/mL and 0-0.8  ng/mL, respectively), although high FSH levels stabilize [4]. There are extensive studies that focus on early-onset menopause, including surgical and spontaneous causes. There is also a specific definition with associated metrics. However, delayed or late-onset menopause is rarely mentioned or reviewed in literature and remains a rare possibility in certain patient groups.

Patients with late-onset menopause or the absence of menopause are predisposed to increased estrogen exposure throughout their lives, potentially elevating their risk of breast and endometrial cancers. Additionally, the increased levels of estrogen may potentially increase a patient’s risk of thrombosis throughout their lifespan.

In our patient, the endometrial stripe was not thickened, and therefore an endometrial biopsy was not performed. Clinical judgement was utilized to determine that no other testing was necessary. The patient was counseled about the possibility of a hysterectomy or some form of surgical menopause secondary to the myoma and the bleeding, but the patient declined. Menstrual symptoms remained well-controlled on NSAIDs as needed for pain. The patient takes aspirin as needed for pain, which could potentially contribute to abnormal uterine bleeding (AUB) and menstrual irregularity [5]. The myoma in the uterus could also contribute to abnormal uterine bleeding due to a structural cause. However, this does not explain the regularity of menstrual cycles. The patient self-reported cycles occurring every 28 days and lasting for about five days, beginning with heavy bleeding for the first three days and light spotting for the final two days.

Additionally, her age of menarche was nine years old, with her two daughters beginning menarche at age eight and nine years old. This adds to the increased lifetime estrogen for this patient, as does the familial tendencies, and to continue, so patients of this nature need to be assessed and followed due to their increased risks of potential high estrogen side effects [6-10].

## Conclusions

Menopause is often utilized as an all-encompassing term and does not account for the variations in causes or ages of onset. Additionally, physicians are not trained to think of a lack of menopause in the geriatric population. Through careful and extensive history, combined with a thorough family history, providers may adjust care for these rare cases to avoid unnecessary extensive testing that may be cost-ineffective or harmful to the patient. Our case serves as an illustrative example that, when combined with existing literature, highlights the importance of considering a newer holistic approach for practitioners evaluating vaginal bleeding in the geriatric population.

## References

[1] Casper RF (2023). Clinical manifestations and diagnosis of menopause. UpToDate.

[2] Cramer DW, Barbieri RL, Xu H, Reichardt JK (1994). Determinants of basal follicle-stimulating hormone levels in premenopausal women. J Clin Endocrinol Metab.

[3] Perkins KE, King MC (2012). Geriatric gynecology. Emerg Med Clin North Am.

[4] Edwards H, Duchesne A, Au AS, Einstein G (2019). The many menopauses: searching the cognitive research literature for menopause types. Menopause.

[5] Walker MH, Coffey W, Borger J (2023). Menorrhagia. StatPearls.

[6] Klein DA, Emerick JE, Sylvester JE, Vogt KS (2017). Disorders of puberty: an approach to diagnosis and management. Am Fam Physician.

[7] Abou-Ismail MY, Citla Sridhar D, Nayak L (2020). Estrogen and thrombosis: a bench to bedside review. Thromb Res.

[8] Treloar AE (1981). Menstrual cyclicity and the pre-menopause. Maturitas.

[9] Prior JC (2006). Perimenopause lost—reframing the end of menstruation. J Reprod Infant Psychol.

[10] Sanderson PA, Critchley HO, Williams AR, Arends MJ, Saunders PT (2017). New concepts for an old problem: the diagnosis of endometrial hyperplasia. Hum Reprod Update.

